# Sedation/Analgesia Administration Practice Varies according to Endoscopy Facility (Hospital- or Office-Based) Setting: Results from a Nationwide Survey in Greece

**DOI:** 10.1155/2020/8701791

**Published:** 2020-10-05

**Authors:** Georgios Tziatzios, Dimitrios N. Samonakis, Theocharis Tsionis, Spyridon Goulas, Dimitrios Christodoulou, Konstantinos Triantafyllou

**Affiliations:** ^1^Hepatogastroenterology Unit, Second Department of Internal Medicine-Propaedeutic, Research Institute and Diabetes Center, Medical School, National and Kapodistrian University of Athens, “Attikon” University General Hospital, Athens, Greece; ^2^Department of Gastroenterology & Hepatology, University Hospital of Heraklion, Crete, Greece; ^3^Gastroenterology Private Practice Facility, Serres, Greece; ^4^Department of Gastroenterology, Elena Venizelou General Hospital, Athens, Greece; ^5^Division of Gastroenterology, University Hospital & Faculty of Medicine, School of Health Sciences, University of Ioannina, Ioannina, Greece

## Abstract

**Objectives:**

To examine the impact of endoscopy setting (hospital-based vs. office-based) on sedation/analgesia administration and to provide nationwide data on monitoring practices among Greek gastroenterologists in real-world settings. *Material and Methods*. A web-based survey regarding sedation/analgesia rates and monitoring practices during endoscopy either in a hospital-based or in an office-based setting was disseminated to the members of the Hellenic Society of Gastroenterology and Professional Association of Gastroenterologists. Participants were asked to complete a questionnaire, which consisted of 35 items, stratified into 4 sections: demographics, preprocedure (informed consent, initial patient evaluation), intraprocedure (monitoring practices, sedative agents' administration rate), and postprocedure practices (recovery).

**Results:**

211 individuals responded (response rate: 40.3%). Propofol use was significantly higher in the private hospital compared to the public hospital and the office-based setting for esophagogastroduodenoscopy (EGD) (85.8% vs. 19.5% vs. 10.5%, *p* < 0.0001) and colonoscopy (88.2% vs. 20.1% vs. 9.4%, *p* < 0.0001). This effect was not detected for midazolam, pethidine, and fentanyl use. Endoscopists themselves administered the medications in most cases. However, a significant contribution of anesthesiology sedation/analgesia provision was detected in private hospitals (14.7% vs. 2.8% vs. 2.4%, *p* < 0.001) compared to the other settings. Only 35.2% of the private offices have a separate recovery room, compared to 80.4% and 58.7% of the private hospital- and public hospital-based facilities, respectively, while the nursing personnel monitored patients' recovery in most of the cases. Participants were familiar with airway management techniques (83.9% with bag valve mask and 23.2% with endotracheal intubation), while 49.7% and 21.8% had received Basic Life Support (BLS) and Advanced Life Support (ALS) training, respectively.

**Conclusion:**

The private hospital-based setting is associated with higher propofol sedation administration both for EGD and for colonoscopy. Greek endoscopists are adequately trained in airway management techniques.

## 1. Introduction

Provision of sedation and analgesia increases gastrointestinal (GI) endoscopy procedural quality and contributes to better patient satisfaction and willingness to undergo endoscopic procedures. Nowadays, sedation use has been optimized, expanding from complex endoscopic procedures, i.e., endoscopic retrograde cholangiopancreatography (ERCP), to everyday routine use in the most commonly performed endoscopic procedures, i.e., esophagogastroduodenoscopy (EGD) and colonoscopy [1]. The advent of efficacious and safe sedative/analgesic medication combinations with rapid onset of action and short-lived effects has revolutionized everyday clinical practice [2]. However, sedation practices in GI endoscopy are not homogenous and may significantly vary, as they must comply each time with local legislation policies and resource availability. Moreover, patient and clinician attitudes that dictate these particular patterns are prone to substantial change over time [3, 4] as well as the setting where endoscopy is carried out [5–7]. However, data comparing sedation practices between hospital-based and office-based settings remain scarce; only a single study reporting on sedation rates between the two settings has been published so far [8]. In this context, we conducted a nationwide survey to investigate the impact of setting on current sedation and monitoring practices during routine GI endoscopy among Greek endoscopists.

## 2. Methods

### 2.1. Study Population and Design

This study was a cross-sectional web survey conducted in February 2020, among gastroenterologists affiliated with the Hellenic Society of Gastroenterology (HSG) and Professional Association of Gastroenterologists (EPEGE). The survey was distributed to endoscopists working in office-based facilities and/or hospitals (public or private).

### 2.2. Development and Administration of Survey Instrument

The survey instrument—a 35-item anonymous questionnaire that could be completed in approximately 8 minutes—was designed by a team of researchers and physicians based on the available literature [8–12], with its final version being approved by the Governing Board of HSG. Subsequently, we used the commercially available version of the web-based survey program “Google Forms” to develop the survey instrument. The survey was distributed using the official HSG and EPEGE databases, by sending individualized e-mail invitations with the link to complete the survey along with a cover letter explaining the purposes of the study. Duplicate participation was prevented by the electronic survey program itself, since only a single answer per user was possible. Responses were automatically recorded in the system and entered into a software database (Microsoft Excel; Microsoft Corp., Redmond, WA, USA), remaining anonymous to study investigators.

### 2.3. Questionnaire

The 35-item questionnaire grouped questions into four distinct sections. The first section (questions Q1–Q10), included questions regarding demographic and professional characteristics of the participants. In the second section (questions Q11–Q14), preendoscopic evaluation, intraprocedural safety equipment use, and patient monitoring practices were assessed. The third section (questions Q15–Q31), comprised questions evaluating sedation practices and attitudes toward sedation, whilst the fourth section (Q32–Q35) assessed respondents' postprocedural practices associated with patients' recovery. The questionnaire is available in the Supplementary Table 1.

### 2.4. Study Endpoints

Primary endpoint

- To examine the impact of setting (hospital-based vs. office-based) on sedation administration during routine GI endoscopy (EGD, colonoscopy)

Secondary endpoints

- To examine the impact of setting (hospital-based vs. office-based) on sedation/recovery practices during routine GI endoscopy (EGD, colonoscopy)
- To assess Greek endoscopists' knowledge level in airway management techniques (performance/training)

### 2.5. Statistical Analysis

Quantitative data are expressed as mean (±SD) and categorical data as number (%). Kolmogorov–Smirnov's statistic was used to assess distribution of quantitative data for normality. Student's *t*-test was used for comparisons of variables with normal distribution, while we used nonparametrical tests to analyze categorical and noncontinuous quantitative variables. For the purposes of our study, we compared only percentages exceeding 75% of use, defined as the majority of cases. All calculations were performed using the software statistical program Statistical Packages for Social Sciences (SPSS) version 25.0 (Chicago, Illinois, USA), with a *p* value < 0.05 considered as significant for all statistical assessments.

### 2.6. Ethical Considerations

The study was approved by the Governing Board of HSG. All participations were voluntary and anonymous. Survey completion did not require registration of potential unique physician identifiers, protecting participants' confidentiality.

## 3. Results

### 3.1. Participants' Characteristics

**Figure 1:**
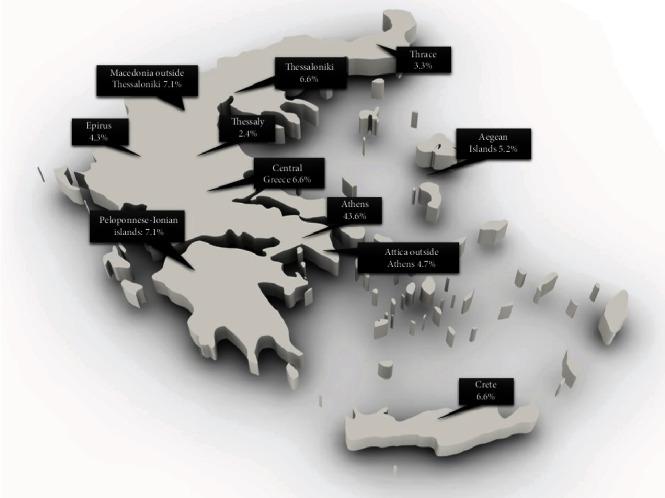
Geographical distribution of participants.

**Figure 2:**
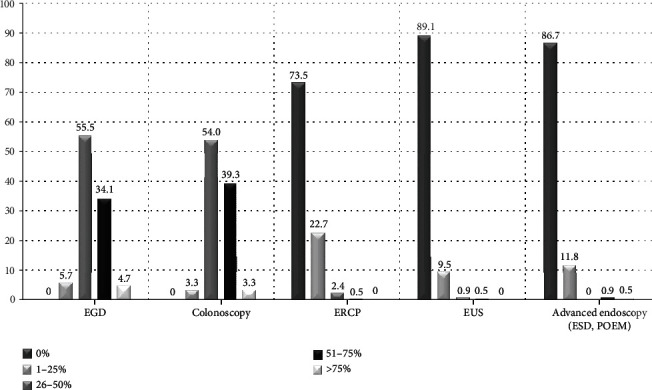
Participants' workload by endoscopic procedure. Data are expressed as percentage according to predefined ranges (0%, 1-25% of procedures, between 26 and 50%, between 51 and 75%, and over 75%). EGD: esophagogastroduodenoscopy; ERCP: endoscopic retrograde cholangiopancreatography; EUS: endoscopic ultrasound; ESD: endoscopic submucosal dissection; POEM: per oral endoscopic myotomy.

**Figure 3:**
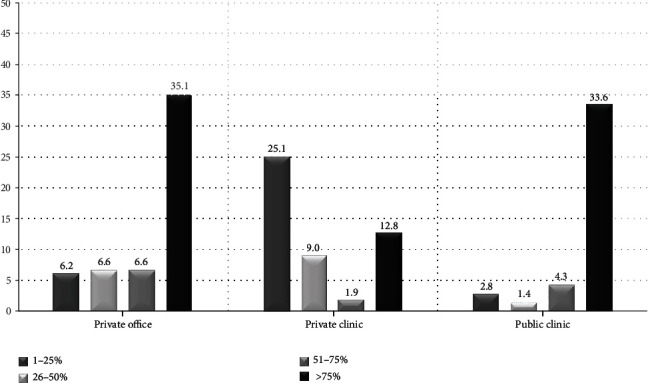
Participants' workload by setting (private office, clinic in private hospital, and clinic in public hospital). Data are expressed as percentage according to predefined ranges (0%, 1-25% of procedures, between 26 and 50%, between 51 and 75%, and over 75%).

In total, 211 members completed the survey online, representing a response rate of 211/524 (40.3%). Participants practiced endoscopy for 12.4 ± 8.6 years. Responses' geographical distribution reflected the population weight of each administrative region in Greece: most results originated from the region of Athens, accounting for almost half (43.6%) of the total sample (Figure 1). As is shown in Figure 2, the majority of respondents performed mainly EGD and colonoscopy during their routine clinical practice. Contrariwise, endoscopic retrograde cholangiopancreatography (ERCP), endoscopic ultrasound (EUS), and advanced endoscopy procedures (endoscopic submucosal dissection (ESD) and per oral endoscopic myotomy (POEM)) comprised only a small proportion of endoscopists' everyday workload. Regarding the working environment, significant variability in the reported results was evident (Figure 3). While some endoscopists worked in multiple settings, 35.1%, 33.6%, and 12.8% of the participants performed >75% of their endoscopic procedures at their private office and public hospital- and private hospital-based facilities, respectively. Overall, 45.5% of the respondents informed their patients in writing about the possible complications in detail and obtained written consent for all procedures, and 10% obtained written consent following written information provision only before high-risk exams. Contrariwise, almost one-fourth (22.3%) of them obtained oral consent from all patients before endoscopy (Table 1).

### 3.2. Study Endpoints

#### 3.2.1. Primary Endpoint

*(1) Sedation in EGD according to Setting*.  Table 2 summarizes sedative/analgesic agents use for EGDs carried out in the two settings (hospital-based vs. office-based). Use of propofol was significantly higher for procedures taking place at a hospital-based environment, compared to the office-based setting (46.3% vs. 7.2%, *p* < 0.0001). Fentanyl use was higher in the office-based setting (28.0% vs. 13.4%, *p* = 0.003), while no difference regarding the use of the other sedative/analgesic agents between the two settings was noted (84.2% vs. 82.0%, *p* = 0.63, for midazolam and 3.0% vs. 5.2%, *p* = 0.38, for pethidine). Restricting the analysis to those performing >75% at each of the different working environments, propofol use was significantly higher within private clinics compared to public ones (85.8% vs. 19.5%, *p* < 0.0001, Table 3) and private offices, as well (85.8% vs. 10.5%, *p* < 0.0001). Fentanyl use did not differ between private and public clinics (25.2% vs. 18.5%, *p* = 0.46) and was also similar between the private clinic and the private office (25.2% vs. 25.7%, *p* = 0.95).

*(2) Sedation in Colonoscopy according to Setting*. Use of propofol was significantly higher for colonoscopies performed at a hospital-based facility, compared to an office-based setting (45.1% vs. 8.8%, *p* < 0.0001, Table 2). Pethidine use was higher for colonoscopies within hospitals, as well (14.2% vs. 4.3%, *p* = 0.009). Midazolam and fentanyl were used at equivalent rates in both settings (90.1% vs. 87.7%, *p* = 0.54, for midazolam and 39.8% vs. 28.7%, *p* = 0.09, for fentanyl). Restricting the analysis to those performing >75% at each of the different working environments, propofol use was significantly higher within private clinics compared to public ones (88.2% vs. 20.1%, *p* < 0.0001, Table 3) and private offices, as well (88.2% vs. 9.4%, *p* < 0.0001). Pethidine use did not differ between private and public clinics (8.2% vs. 24.9%, *p* = 0.08) and was similar also between the private clinic and the private office (8.2% vs. 6.2%, *p* = 0.73).

#### 3.2.2. Secondary Endpoints

**Figure 4:**
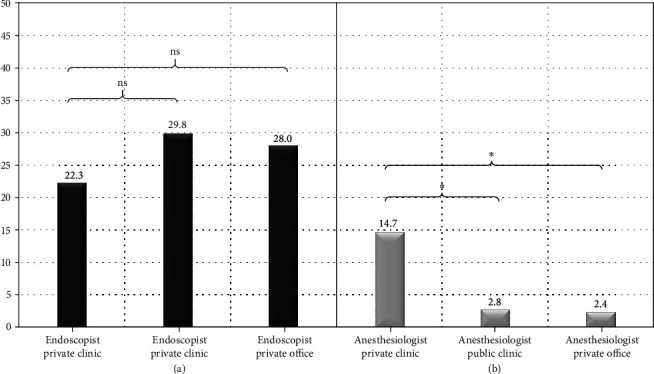
Sedation administration of (a) endoscopist and (b) anesthesiologist, by setting (clinic in private hospital, clinic in public hospital, and private office). ns: not significant; ^∗^*p* < 0.05.

*(1) Sedation Administration*. The endoscopist administered sedatives/analgesic in most of the cases; no difference regarding frequency among the three settings was found (22.3% vs. 29.8%, 28.0%, *p* = 0.09, Figure 4(a)). On the contrary, an anesthesiologist was involved in the medications' administration significantly more frequently in a private hospital-based facility as compared to the two other settings (14.7% vs. 2.8%, 2.4%, *p* < 0.0001, Figure 4(b)). All the respondents' monitored vital signs and pulse oximetry, while capnography was rarely used (7.6%), irrespective of the working environment.

*(2) Patients' Recovering Practices*. Almost half of the participants (99/211, 46.9%) stated that patients recover under the monitoring of the nursing personnel, while 92 (43.6%) of them monitored patients after the endoscopic procedure themselves. Twenty (9.5%) physicians acknowledged the anesthesiologist as responsible. Only thirty-five percent (51/211, 35.2%) of the private offices have a separate recovery room, while this number considerably increases (80.4%) when hospital-based facilities are concerned.

*(3) Airway Management Techniques Proficiency Level and Training*. The vast majority of the participants were familiar with airway management techniques, in particular, bag valve mask (BVM) use (83.9%), while 23.2% of them reported to be familiar with endotracheal intubation (Table 2). As far as training is concerned, half of the respondents (49.7%) had attended a cardiopulmonary resuscitation Basic Life Support (BLS) course and one out of five (21.8%) had been awarded Advanced Life Support (ALS) certified training. During the patient's initial assessment, endoscopists' were familiar with sedation/anesthesia levels as well as the American Society of Anesthesiologists (ASA) physical status classification system. On the contrary, Mallampati's classification was seldom used (3.8%).

## 4. Discussion

Results of this first web-based nationwide survey among Greek gastroenterologists provide valuable data regarding the impact of endoscopy setting (hospital-based vs. office-based) on sedation/analgesia administration during routine GI endoscopy. To date, only one study has addressed this issue [8], reporting higher hospital-based sedation rate compared to office setting (89.7% vs. 86.4%; *p* < 0.0001), but only as a secondary outcome.

Our analysis quantified propofol implementation in clinical practice, demonstrating that its use was significantly higher in the private hospital-based setting, regardless the procedure performed. Interpreting this result can be challenging and speculative; an explanation could be offered, based on the fact that sedation during endoscopy within a Greek hospital environment is directed by an anesthesiologist, which is the only medical specialty accredited by regional legal regulations to administer propofol. However, the availability of anesthesiologists in the Greek public hospital is limited; thus, their work outside the operating rooms is restricted to complex, advanced, and time-consuming procedures. On the other hand, nonanesthesiologist (gastroenterologist-directed) administration of propofol remains prohibited; thus, administering propofol within private office practice requires the presence of an anesthesiologist, translating into additional financial burden, at a time when local payer policies regarding anesthesia service coverage in GI endoscopy may also vary. Additionally, it might represent a shift in endoscopists' attitude towards sedation. Not only have they mastered their skills in sedation over the years, being able to deliver it safely, but they also use it because they consider it beneficial. Interestingly, fentanyl use was not related to endoscopy facility setting. Since fentanyl administration is also restricted to anesthesiologists, this finding can hardly been explained.

Complementary to the above, the vast majority of respondents (92%) indicated that sedation administration was administered mainly by the endoscopist. Indeed, worldwide, this finding seems to be perhaps one of the few that are common among all studies reporting on sedation in GI endoscopy [11, 13, 14]. Although variations in the rate of contribution can be noted, sedation administration is still predominantly performed by endoscopists, a practice that remained unaltered in the course of time also for Greece [9, 10]. However, an increased participation of anesthesiologists in the provision of sedation/analgesia has been noted in private hospitals, and this can obviously be related to the findings of our study primary outcome. Of note, endoscopists also supervised the patient's recovery in almost half of the cases (43.6%), with the nursing personnel being also equally involved (46.9%) in this part.

Our study raises for the first time a number of meaningful sedation quality implications directly related to endoscopists' level of training and patients' vigilance, as well. One of the most interesting findings was that a large group of respondents is familiar with effective airway management (mask ventilation in 83.9%, endotracheal intubation in 23.2%), even surpassing the results reported internationally [14]. Respiratory failure remains the cardinal life-threatening sedation-derived complication, regardless the type of sedative or analgesic used [2, 15]. In this sense, Greek gastroenterologists display a high level of professionalism, being adequately trained in dealing with airway management techniques, as this is the first step to guarantee patient safety during endoscopic sedation in the pursuit of optimizing the quality of delivered services. This is further corroborated by the fact the vast majority of participants had attended at least one patients' resuscitation official course (BLS, ALS, and ILS). Despite being mandatory in some European countries, participation to these training programs in Greece remains at each physician's discreet choice, with their expenses covered by themselves. To meet this unprecedented demand, EPEGE responded promptly by launching a preeminent initiative, providing its members ILS training courses yearly, at no cost.

Furthermore, monitoring patterns were uniform among survey participants, involving heart rate and pulse oximetry monitoring in all cases, while capnography was seldom used. This finding clearly delineates their adherence to established guidelines [16]. The fact that half of the respondents informed their patients in writing about possible complications and obtained written consent for all procedures, while 10% of them obtain written consent only for high-risk procedures, remains indeed suboptimal, leaving room for improvement prior to reaching the desirable performance target [17]. Of note, no other similar study to date has reported on this outcome, constituting any comparison impossible. Perhaps more ominous is that only a fragment of participants (25.1%) incorporates the patient's American Society of Anesthesiologists (ASA) physical status assessment prior to each procedure, and to make things even more challenging, Greek endoscopists do not use the Mallampati classification [18]. Our European colleagues seem to perform better in this field (ASA: 47.8%/Mallampati classification: 18.6%) [11]; however, their performance is also far from perfect yet [16, 19]. In light of these observations, use of preendoscopy risk assessment methods and rating scales as advocated by current guidelines [16, 19] should be integrated in endoscopists' structural and personal requirements that are essential for sedation in gastrointestinal endoscopy.

The core strength of our study is its novelty; to the best of our knowledge, this is the first iteration evaluating the impact of endoscopy's setting in sedation practice. Second is the representativeness of our sample, since we contacted and collected data from all members of our national scientific and professional societies. Third, all available GI endoscopy settings (private office, public, or private clinic) were analyzed. Finally, we achieved a response rate of 40.3%, which not only is equivalent to response rates previously reported locally [9, 10], but also exceeds that reported in recent surveys (26%), carried out within country with a population similar to ours [20].

Our study's principal limitation relates to the study's inherent design, since this type of studies is per se susceptible to recall and self-report bias.

In summary, our national survey provides evidence supporting the notion that a private hospital-based setting is associated with higher propofol sedation rates both for EGD and for colonoscopy. Data acquired may be used in promoting initiatives to improve medical care and quality targets, regarding sedation/analgesia provision for routine endoscopic procedures.

## Figures and Tables

**Table 1:** Survey responses.

Question		*N* (%)
*Preprocedure practices*		

Informed consent before the endoscopic procedure (*n* = 211)	I inform in writing about the possible complications in detail, and I get written consent from all patients	96 (45.5)
	I do not receive written informed consent, but I inform about possible complications in detail, and I get oral consent from all patients	47 (22.3)
	I do not receive written informed consent, but I do inform about possible complications in detail, and I receive oral consent from patients undergoing diagnostic procedures	42 (19.9)
	I inform in writing about the possible complications in detail, and I get written consent only in high-risk procedures	21 (10.0)
	I inform in writing about the possible complications, and I get written consent from all patients for endoscopy without sedation	4 (1.9)
	I inform in detail, and I get written consent for the possibility of endoscopy without sedation	1 (0.5)

Endoscopist administering intravenous sedation is familiar with (*n* = 211)^∗^	Airway management techniques	183 (86.7)
	Oropharyngeal airway management	138 (65.4)
	Bag valve mask- (BVM-) Ambu use	177 (83.9)
	Endotracheal intubation	49 (23.2)
	Basic Life Support (BLS)	105 (49.7)
	Advanced Life Support (ALS)	46 (21.8)
	Immediate Life Support (ILS)	113 (53.6)

During initial assessment of the patient, endoscopist is familiar with (*n* = 211)	Sedation/anesthesia levels	94 (44.5)
	ASA physical status classification system and sedation/anesthesia levels	53 (25.1)
	All the above	26 (12.3)
	American Society of Anesthesiologists (ASA) physical status classification system	22 (10.4)
	The Mallampati score to predict difficult intubation	8 (3.8)
	The Mallampati score and sedation/anesthesia levels	6 (2.8)
	ASA physical status classification system and the Mallampati score	2 (0.9)

*Intraprocedure practices*		

Monitoring of patient during examination by (*n* = 211)^∗^	SaO_2_ and pulse monitoring	211 (100.0)
	*Α*rterial blood pressure monitoring	108 (51.1)
	Electrocardiogram monitoring	44 (20.9)
	Capnography monitoring	16 (7.6)

Who is the sedation administrator during endoscopy? (*n* = 211)	At the private office, the endoscopist	59 (28.0)
	At the private office, the anesthesiologist	5 (2.4)
	At the private clinic, the endoscopist	47 (22.3)
	At the private clinic, the anesthesiologist	31 (14.7)
	At the public hospital, the endoscopist	63 (29.9)
	At the public hospital, the anesthesiologist	6 (2.8)

*Postprocedure practices*		

Patient's resuscitation is performed (*n* = 211)	Under supervision of the nursing personnel, in most cases	99 (46.9)
	Under supervision of the endoscopist, in most cases	92 (43.6)
	Under supervision of the anesthesiologist, in most cases	20 (9.5)

Separate resuscitation room	Private office (*n* = 145)	51 (35.2)
	Private clinic (*n* = 102)	82 (80.4)
	Public clinic (*n* = 92)	54 (58.7)

*n*: number of responses received for each question. ^∗^Sum is greater than 100% due to multiple possible answers for the question.

**Table 2:** Comparison of sedative/analgesic agents' use between private office and hospital-based (private and public) settings.

	0%		1–25%		26–50%		51–75%		>75%		*p*
	Office setting	Hospital setting	Office setting	Hospital setting	Office setting	Hospital setting	Office setting	Hospital setting	Office setting	Hospital setting	
*EGD*											

Midazolam	0%	5.6%	7.1%	2.5%	5.8%	4.3%	2.9%	5.6%	84.2%	82.0%	*p* = 0.63
Pethidine	95.5%	78.7%	1.5%	12.3%	0%	1.9%	0%	1.9%	3.0%	5.2%	*p* = 0.38
Fentanyl	67.6%	69.4%	1.5%	7.0%	1.5%	2.5%	1.4%	7.6%	28.0%	13.4%	*p* = 0.003
Propofol	88.4%	34.1%	1.6%	9.8%	1.4%	7.3%	1.4%	2.4%	7.2%	46.3%	*p* < 0.0001

*Colonoscopy*											
Midazolam	7.1%	6.2%	0%	0%	1.4%	3.7%	1.4%	2.5%	90.1%	87.7%	*p* = 0.54
Pethidine	89.9%	66.5%	4.4%	9.7%	1.4%	9.0%	0%	0.6%	4.3%	14.2%	*p* = 0.009
Fentanyl	52.9%	46.9%	4.4%	13.8%	0%	6.3%	2.9%	4.4%	39.8%	28.7%	*p* = 0.09
Propofol	83.8%	31.5%	4.4%	9.9%	3.0%	8.6%	0%	4.9%	8.8%	45.1%	*p* < 0.0001

EGD: esophagogastroduodenoscopy.

**Table 3:** Comparison of sedative/analgesic agents' use between private office and hospital-based (private and public) settings when workload > 75%.

Medication	Private clinic	Public clinic	*p* ^∗^	Private office	*p* ^∗∗^
*EGD*					

Midazolam	78.7%	89.1%	*p* = 0.18	87.3%	*p* = 0.29
Pethidine	6.2%	12.4%	*p* = 0.44	2.3%	*p* = 0.38
Fentanyl	25.2%	18.5%	*p* = 0.46	25.7%	*p* = 0.95
Propofol	85.8%	19.5%	*p* < 0.0001	10.5%	*p* < 0.0001

*Colonoscopy*					

Midazolam	81.1%	93.9%	*p* = 0.06	87.5%	*p* = 0.41
Pethidine	8.2%	24.9%	*p* = 0.08	6.2%	*p* = 0.73
Fentanyl	31.6%	32.2%	*p* = 0.95	40.1%	*p* = 0.45
Propofol	88.2%	20.1%	*p* < 0.0001	9.4%	*p* < 0.0001

^∗^ *p* value referring to the comparison between private and public clinic. ^∗∗^*p* value referring to the comparison between private clinic and private office. EGD: esophagogastroduodenoscopy.

## Data Availability

All data used to support the findings of this study are included within the article and also available from the corresponding author upon request.

## Supplementary Materials

Supplementary Materials Supplementary Table 1: questionnaire used.
