# Supplementary material for: Sedation/Analgesia Administration Practice Varies according to Endoscopy Facility (Hospital- or Office-Based) Setting: Results from a Nationwide Survey in Greece
Source: Gastroenterol Res Pract. 2020 Oct 5;2020:8701791. doi: 10.1155/2020/8701791 (PMC7556063; doi:10.1155/2020/8701791)
Supplement: Supplementary Materials — Supplementary Table 1: questionnaire used. [file 8701791.f1.docx]

**Appendix A.** Questionnaire used

| **Section A. Demographics**  *Please answer the following questions by choosing the most appropriate answer* | | | | | | | | | | | | | | | | | | | | | | |
| --- | --- | --- | --- | --- | --- | --- | --- | --- | --- | --- | --- | --- | --- | --- | --- | --- | --- | --- | --- | --- | --- | --- |
| Q1 | How many years do you practice endoscopy? |  | | | | | | | | | | | | | | | | | | | | |
| Q2 | In which geographical region do you perform endoscopy? | Athens | | Attica outside Athens | | | | Thessaloniki | | | | | | Macedonia outside Thessaloniki | | | | Central Greece | | | | Epirus |
|  | | Thessaly | | Thrace | | | | Peloponnesus | | | | | | Crete | | | | Ionian Islands | | | | Aegean Islands |
| Q3 | Which percentage of your total endoscopic workload includes Esophagogastroduodenoscopy (EGD) performance? | 0% | | | | | 1 – 25% | | | | | | 26 – 50% | | | | 51 – 75% | | | | >75% | |
| Q4 | Which percentage of your endoscopic workload includes colonoscopy performance? | 0% | | | | | 1 – 25% | | | | | | 26 – 50% | | | | 51 – 75% | | | | >75% | |
| Q5 | Which percentage of your endoscopic workload includes Endoscopic Retrograde Cholangio - Pancreatography (ERCP) performance? | 0% | | | | | 1 – 25% | | | | | | 26 – 50% | | | | 51 – 75% | | | | >75% | |
| Q6 | Which percentage of your endoscopic workload includes Endoscopic ultrasound (EUS) performance? | 0% | | | | | 1 – 25% | | | | | | 26 – 50% | | | | 51 – 75% | | | | >75% | |
| Q7 | Which percentage of your endoscopic workload includes performance of advanced Endoscopy procedures (Endoscopic submucosal dissection - ESD, Per oral endoscopic myotomy - POEM)? | 0% | | | | | 1 – 25% | | | | | | 26 – 50% | | | | 51 – 75% | | | | >75% | |
| Q8 | Which percentage of your endoscopic workload includes working in a Private Office? | 0% | | | | | 1 – 25% | | | | | | 26 – 50% | | | | 51 – 75% | | | | >75% | |
| Q9 | Which percentage of your total endoscopic workload includes working in a Private Clinic? | 0% | | | | | 1 – 25% | | | | | | 26 – 50% | | | | 51 – 75% | | | | >75% | |
| Q10 | Which percentage of your endoscopic workload includes working in the National Healthcare System (i.e. public hospitals)? | 0% | | | | | 1 – 25% | | | | | | 26 – 50% | | | | 51 – 75% | | | | >75% | |
| **Section B. Pre - endoscopic evaluation, intra-procedural safety equipment and patient monitoring practices**  *Please answer the following questions by choosing the most appropriate answer(s)* | | | | | | | | | | | | | | | | | | | | | | |
| Q11 | Which of the following practices do you use to obtain informed consent regarding sedation before performing the endoscopic procedure? (more than one answer possible) | I do not receive written informed consent, but I inform about possible complications in detail and I get oral consent from all patients | | | | | I do not receive written informed consent, but I do inform about possible complications in detail and I receive oral consent from patients undergoing diagnostic procedures | | | | | | I inform in writing about the possible complications in detail and I get written consent from all patients | | | | I inform in writing about the possible complications in detail and I get written consent only in high risk procedures | | | | I inform in detail and I get written consent for the possibility of endoscopy without sedation | |
| Q12 | As an endoscopist who administers intravenous sedation, you are familiar with the following (more than one answer possible): | Airway management techniques | Oropharyngeal airway management | | | | Bag valve mask (BVM) - Ambu - use | | | Endotracheal intubation | | | Basic Life Support (BLS) | | | | Advanced Life Support (ALS) | | | | Immediate Life Support (ILS) | |
| Q13 | As part of the initial assessment of the patient who will receive sedation, you are familiar with (more than one answer possible): | The Mallampati score to predict difficult intubation | | | | | American Society of Anesthesiologists (ASA) Physical Status Classification System | | | | | | | | | | Sedation and anesthesia levels | | | | | |
| Q14 | In your endoscopic practice, what type of monitoring do you necessarily use in each examination (more than one answer possible)? | SaO_2_ and pulses monitoring | | | | | Arterial Blood pressure monitoring | | | | Electrocardiogram monitoring | | | | | | Capnography | | | | | |
| **Section C***.* Sedation practices  *Please answer the following questions by choosing the most appropriate answer* | | | | | | | | | | | | | | | | | | | | | | |
| Q15 | In your endoscopic practice at the Private Office, to what percentage do you use ***Midazolam*** during Esophagogastroduodenoscopy (EGD)? If you do not have a private office, please proceed below | 0% | | | 1 – 25% | | | | 26 – 50% | | | | | | 51 – 75% | | | | | >75% | | |
| Q16 | In your endoscopic practice at the Private Office, to what percentage do you use ***Pethidine*** during Esophagogastroduodenoscopy (EGD)? If you do not have a private office, please proceed below | 0% | | | 1 – 25% | | | | 26 – 50% | | | | | | 51 – 75% | | | | | >75% | | |
| Q17 | In your endoscopic practice at the Private Office, to what percentage do you use ***Fentanyl*** during Esophagogastroduodenoscopy (EGD)? If you do not have a private office, please proceed below | 0% | | | 1 – 25% | | | | 26 – 50% | | | | | | 51 – 75% | | | | | >75% | | |
| Q18 | In your endoscopic practice at the Private Office, to what percentage do you use ***Propofol*** during Esophagogastroduodenoscopy (EGD)? If you do not have a private office, please proceed below | 0% | | | 1 – 25% | | | | 26 – 50% | | | | | | 51 – 75% | | | | | >75% | | |
| Q19 | In your endoscopic practice at the Private Office, to what percentage do you use ***Midazolam*** during Colonoscopy? If you do not have a private office, please proceed below | 0% | | | 1 – 25% | | | | 26 – 50% | | | | | | 51 – 75% | | | | | >75% | | |
| Q20 | In your endoscopic practice at the Private Office, to what percentage do you use ***Pethidine*** during Colonoscopy? If you do not have a private office, please proceed below | 0% | | | 1 – 25% | | | | 26 – 50% | | | | | | 51 – 75% | | | | | >75% | | |
| Q21 | In your endoscopic practice at the Private Office, to what percentage do you use ***Fentanyl*** during Colonoscopy? If you do not have a private office, please proceed below | 0% | | | 1 – 25% | | | | 26 – 50% | | | | | | 51 – 75% | | | | | >75% | | |
| Q22 | In your endoscopic practice at the Private Office, to what percentage do you use ***Propofol*** during Colonoscopy? If you do not have a private office, please proceed below | 0% | | | 1 – 25% | | | | 26 – 50% | | | | | | 51 – 75% | | | | | >75% | | |
| Q23 | In your endoscopic practice at the Clinic (public or private), to what percentage do you use ***Midazolam*** during Esophagogastroduodenoscopy (EGD)? If you perform endoscopy only in a private office, please proceed below | 0% | | | 1 – 25% | | | | 26 – 50% | | | | | | 51 – 75% | | | | | >75% | | |
| Q24 | In your endoscopic practice at the Clinic (public or private), to what percentage do you use ***Pethidine*** during Esophagogastroduodenoscopy (EGD)? If you perform endoscopy only in a private office, please proceed below | 0% | | | 1 – 25% | | | | 26 – 50% | | | | | | 51 – 75% | | | | | >75% | | |
| Q25 | In your endoscopic practice at the Clinic (public or private), to what percentage do you use ***Fentanyl*** during Esophagogastroduodenoscopy (EGD)? If you perform endoscopy only in a private office, please proceed below | 0% | | | 1 – 25% | | | | 26 – 50% | | | | | | 51 – 75% | | | | | >75% | | |
| Q26 | In your endoscopic practice at the Clinic (public or private), to what percentage do you use ***Propofol*** during Esophagogastroduodenoscopy (EGD)? If you perform endoscopy only in a private office, please proceed below | 0% | | | 1 – 25% | | | | 26 – 50% | | | | | | 51 – 75% | | | | | >75% | | |
| Q27 | In your endoscopic practice at the Clinic (public or private), to what percentage do you use ***Midazolam*** during Colonoscopy? If you perform endoscopy only in a private office, please proceed below | 0% | | | 1 – 25% | | | | 26 – 50% | | | | | | 51 – 75% | | | | | >75% | | |
| Q28 | In your endoscopic practice at the Clinic (public or private), to what percentage do you use ***Pethidine*** during Colonoscopy? If you perform endoscopy only in a private office, please proceed below | 0% | | | 1 – 25% | | | | 26 – 50% | | | | | | 51 – 75% | | | | | >75% | | |
| Q29 | In your endoscopic practice at the Clinic (public or private), to what percentage do you use ***Fentanyl*** during Colonoscopy? If you perform endoscopy only in a private office, please proceed below | 0% | | | 1 – 25% | | | | 26 – 50% | | | | | | 51 – 75% | | | | | >75% | | |
| Q30 | In your endoscopic practice at the Clinic (public or private), to what percentage do you use ***Propofol*** during Colonoscopy? If you perform endoscopy only in a private office, please proceed below | 0% | | | 1 – 25% | | | | 26 – 50% | | | | | | 51 – 75% | | | | | >75% | | |
| Q31 | Who administers the sedation agents during endoscopy (more than one answer possible)? | At the private office always the endoscopist | | | | At the private office always the anesthesiologist | | | | At the private Clinic always the anesthesiologist | | | | | At the public hospital always the endoscopist | | | | At the public hospital the endoscopist in majority of cases | | | |
|  |  | At the private office the endoscopist in majority of cases | | | | At the private Clinic always the endoscopist | | | | At the private clinic the endoscopist in majority of cases | | | | | At the public hospital always the anesthesiologist | | | |  | | | |
| **Section D***.* **Post - procedural practices associated with patients’ resuscitation**  *Please answer the following questions by choosing the most appropriate answer(s)* | | | | | | | | | | | | | | | | | | | | | | |
| Q32 | Patient’s recovery is performed: | Under supervision of the endoscopist, in most cases | | | | | | | | Under supervision of the nursing personnel, in most cases | | | | | | Under supervision of the anesthesiologist, in most cases | | | | | | |
| Q33 | Is there a separate patient’s resuscitation room in the Private Office you are working? Please answer according to your clinical practice | Yes | | | | | | | | | | No | | | | | | | | | | |
| Q34 | Is there a separate patient’s resuscitation room in the Private Clinic you are working? Please answer according to your clinical practice | Yes | | | | | | | | | | No | | | | | | | | | | |
| Q35 | Is there a separate patient’s resuscitation room in the Public Clinic you are working? Please answer according to your clinical practice | Yes | | | | | | | | | | No | | | | | | | | | | |
